# Extended Spectrum Beta-Lactamase *Escherichia coli* in River Waters Collected from Two Cities in Ghana, 2018–2020

**DOI:** 10.3390/tropicalmed6020105

**Published:** 2021-06-20

**Authors:** Regina Ama Banu, Jorge Matheu Alvarez, Anthony J. Reid, Wendemagegn Enbiale, Appiah-Korang Labi, Ebenezer D. O. Ansa, Edith Andrews Annan, Mark Osa Akrong, Selorm Borbor, Lady A. B. Adomako, Hawa Ahmed, Mohammed Bello Mustapha, Hayk Davtyan, Phillip Owiti, George Kwesi Hedidor, Gerard Quarcoo, David Opare, Boi Kikimoto, Mike Y. Osei-Atwenebanoa, Heike Schmitt

**Affiliations:** 1 CSIR-Water Research Institute, Council for Scientific and Industrial Research, 2nd CSIR Close, Achimota, Accra P.O. Box AH 38, Ghana; markosaakrong@gmail.com (M.O.A.); borborviich.sb@gmail.com (S.B.); asantewa84@gmail.com (L.A.B.A.); hawaahmed360@yahoo.com (H.A.); yarbello@yahoo.com (M.B.M.); gquarcoo@csir-water.com (G.Q.); oseiatweneboana@yahoo.co.uk (M.Y.O.-A.); 2 Department of Surveillance Prevention and Control of AMR, AMR Division, World Health Organization, Route de Sauverny 22, 1290 Versoix, Switzerland; matheujo@who.int; 3 Operational Research Unit (LuxOR), Medical Department, Médecins Sans Frontières Operational Centre Brussels, L-1617 Luxembourg, Luxembourg; tony.reid@brussels.msf.org; 4 Dermatovenereology Department, BahirDar Department University, BahirDar P.O. Box 1996, Ethiopia; wendemagegnenbiale@gmail.com; 5 Amsterdam UMC, Academic Medical Centre, Department of Dermatology, Amsterdam Institute for Infection and Immunity (AI&I), University of Amsterdam, 1012 WX Amsterdam, The Netherlands; 6 WHO Country Office, Ghana, 7 Ameda Street, Roman Ridge, Accra P.O. Box MB 142, Ghana; labia@who.int (A.-K.L.); hedidorg@who.int (G.K.H.); 7 Council for Scientific and Industrial Research-Animal Research Institute, Adenta-Frafraha, Achimota-Accra P.O. Box AH 20, Ghana; edoansa@yahoo.com; 8 World Health Organization, Harare, Zimbabwe; andrewse@who.int; 9 Tuberculosis Research and Prevention Center NGO, Yerevan 0040, Armenia; haykdav@gmail.com; 10 The International Union against Tuberculosis and Lung Disease, 68 Boulevard Saint Michel, 75006 Paris, France; philip.owiti@theunion.org; 11 National Public Health & Reference Laboratory Ghana Health Service, Accra P.O. Box 300, Ghana; opared_60@yahoo.co.uk; 12 National Food Safety/AMR Reference Laboratory for Animal Health (Terrestrial & Aquatic Animals), Veterinary Services, Accra P.O. Box M 161, Ghana; boikikimoto@gmail.com; 13 Centre for Zoonoses and Environmental Microbiology, Centre for Infectious Disease Control, National Institute for Public Health and the Environment (RIVM), P.O. Box 1, 3720 BA Bilthoven, The Netherlands; heike.schmitt@rivm.nl; 14 WHO Collaborating Centre for Risk Assessment of Pathogens in Food and Water at the National Institute for Public Health and the Environment (RIVM), P.O. Box 1, 3720 BA Bilthoven, The Netherlands

**Keywords:** ESBL-*E. coli*, environment, antimicrobial resistance, rivers, Ghana, tricycle protocol, operational research, sort it

## Abstract

Infections by Extended-Spectrum Beta-Lactamase producing *Escherichia coli* (ESBL-Ec) are on the increase in Ghana, but the level of environmental contamination with this organism, which may contribute to growing Antimicrobial Resistance (AMR), is unknown. Using the WHO OneHealth Tricycle Protocol, we investigated the contamination of *E. coli* (Ec) and ESBL-Ec in two rivers in Ghana (Odaw in Accra and Okurudu in Kasoa) that receive effluents from human and animal wastewater hotspots over a 12-month period. Concentrations of Ec, ESBL-Ec and percent ESBL-Ec/Ec were determined per 100 mL sample. Of 96 samples, 94 (98%) were positive for ESBL-Ec. concentrations per 100 mL (MCs100) of ESBL-Ec and %ESBL-Ec from both rivers were 4.2 × 10^4^ (IQR, 3.1 × 10^3^–2.3 × 10^5^) and 2.79 (IQR, 0.96–6.03), respectively. MCs100 were significantly lower in upstream waters: 1.8 × 10^4^ (IQR, 9.0 × 10^3^–3.9 × 10^4^) as compared to downstream waters: 1.9 × 10^6^ (IQR, 3.7 × 10^5^–5.4 × 10^6^). Both human and animal wastewater effluents contributed to the increased contamination downstream. This study revealed high levels of ESBL-Ec in rivers flowing through two cities in Ghana. There is a need to manage the sources of contamination as they may contribute to the acquisition and spread of ESBL-Ec in humans and animals, thereby contributing to AMR.

## 1. Introduction

The rising problem of Antimicrobial Resistance (AMR) is well recognised as a major public health threat [1,2]. The health and economic consequences of AMR are severe, particularly in low and middle-income countries (LMICs) [3,4]. The increase in AMR is widely attributed to inappropriate use of antimicrobials in humans and animal husbandry [5,6] with a resulting impact on the environment, which may, in turn, be a source for the acquisition and transfer of resistant genes. 

Available studies on AMR and the environment are predominantly from high resource settings [7,8,9], with a gap in knowledge from low and middle-income settings [10,11]. AMR is facilitated by the discharge of poorly treated human and animal waste as well as antimicrobials from pharmaceutical industries and health facilities into the environment [12,13]. Antimicrobial-resistant organisms in the environment may then be transmitted to humans through the water supply and food chain [4]. The health of people connects to the health of the environment and AMR data from the environment will be useful in addressing the AMR problem in Ghana.

Extended-spectrum beta-lactamase (ESBL) producing organisms are resistant to important β-lactam antibiotics, which are broad-spectrum antimicrobials commonly used to manage infections [4,12]. They are also most often resistant to other classes of antibiotics thus leaving fewer treatment options when infections occur [10,14].

In Ghana, various studies have shown that the burden of ESBL infections is on the increase, along with community carriage [15,16,17]. Although there is evidence to show that environmental sources may serve as a potential source of transmission of ESBL producing organisms [18,19,20], there are currently no studies on the presence of ESBL-producing organisms in water sources in Ghana. In a broader context, data on presence of ESBL-producing entero-bacteriaceae from the whole continent of Africa are extremely scarce. The few existing studies have shown the presence of ESBL-producing entero-bacteriaceae in sachet drinking water and wastewater (the Democratic Republic of Congo), in untreated wastewater, contaminated surface and groundwater [21], and in poultry and poultry environments [22] (Nigeria), highlighting that prevention of ESBL transmission through drinking water and surface water requires attention. This is because surface water in African urban environments, with open drains, is highly contaminated with faecal organisms [23].

*Escherichia coli* (*E. coli*) is used as an indicator of faecal contamination of water bodies when analyzing water [24]. The presence of Extended Spectrum Beta-Lactamase *E. coli* (ESBL-Ec) could then be also considered as an indicator for antimicrobial resistance in water sources [25,26].

In AMR surveillance studies, the spotlight has remained on humans and animals perhaps due to the difficulties in enumerating pathogens from the environment, use of insufficiently sensitive analytical methods [27] or a lack of sufficient awareness of the environment’s role in the transfer of resistance genes. Quantification of variables such as concentration of ESBL-*E. coli* (ESBL-Ec) and proportions of ESBL-Ec among *E. coli* from various water types, for instance, could provide baseline data for future comparison. The World Health Organizations’ Tricycle Protocol for surveillance of ESBL-Ec in the environment demonstrates the use of these variables in various water types [28]. This protocol has been piloted and found useful in LMICs (Pakistan, Jordan, Indonesia, Nepal, India, Malaysia and Madagascar) [25,29], including Ghana. In these piloting countries, goals were to establish feasibility of the protocol and demonstrate increased inter-institutional collaboration upon implementation of the protocol.

This study aimed to systematically gather information on the extent and origin of environmental contamination of surface water by ESBL-Ec. Specifically, this was achieved through applying the WHO Tricycle protocol for determining counts of *E. coli* and ESBL-Ec in upstream and downstream water of two river bodies, contaminated with animal and human wastewater located in Kasoa and Accra, Southern Ghana, for twelve months. We interpret the findings with respect to the feasibility of the WHO Tricycle protocol and with respect to the degree of environmental contamination.

## 2. Materials and Methods

### 2.1. Study Design

This is a cross-sectional, descriptive study using data from the pilot study of the Tricycle Protocol in Ghana from 2018 to 2020.

### 2.2. Study Setting

#### 2.2.1. General Setting

**Figure 1:**
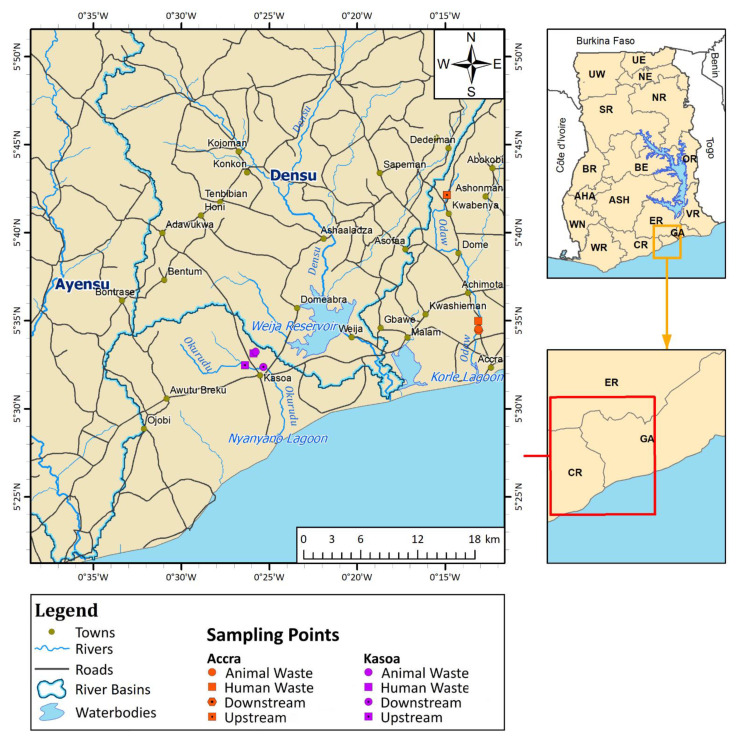
Map showing sampling points from Accra and Kasoa, for the periods of August 2018–December 2019, October 2019–February 2020, May and June 2020.

Ghana is located in West Africa (see Figure 1) with a population of about 30 million [30]. The study site falls within the Coastal River Basin with an average annual rainfall of 800 mm. It is the most urbanised basin in the country and is frequently contaminated by discharge from untreated waste sources and leachates from agrochemicals [31].

#### 2.2.2. Specific Setting: Accra and Its Sampling Locations

Accra is the capital city of Ghana and is located in the south on the Atlantic coast. It has an estimated population of 2.5 million [32] with a sewage management plan that covers approximately 30% of the sewage generated. Accra has four sewage treatment plants, with approximately 15% of the total land area of Accra’s central business district connected to a sewer network [33]. Seven percent of urban dwellers resort to open defecation [34,35]. Household sanitation coverage is approximately 28%, with 72% of households using shared sanitation facilities [35].

The Odaw River is a major river that courses through Accra. It takes its source from the Akwapim Mountain range, runs through Kwabenya, a suburb of Accra, and a centralised open drain in Avenor. It finally empties into Korle lagoon, an outlet into the Atlantic Ocean.

Avenor, a popular catchment area around the Odaw River, is a light industrial area and has a major transport terminal linking Accra to many parts of the country. Surrounding these terminals are shopping centres and an open market characterised by heavy human activity. There are slums in this area known for their open defecation practices along the river. Upstream water samples from the Odaw River were taken from Kwabenya, located 23 km to the north of Avenor, where downstream samples were collected. Human wastewater was collected from a runoff point into the Odaw River whilst animal wastewater samples were taken from a drain outlet from a major slaughter slab (that deals in cattle, sheep and goats). The sampling points are shown on the map in Figure 1.

#### 2.2.3. Specific Setting: Kasoa and Its Sampling Locations

Kasoa is a fast-growing peri-urban township in the Central Region of Ghana with a projected population of over 100,000. It is located further west, away from Accra. The master plan of Accra demarcated Kasoa to remain part of its green belt [36]. As a result, it did not have an original township development plan and has no centralised means of managing its waste. The community mostly uses shared public and household toilets. Kasoa discharges about 14% (246 m^3^/day) of its total faecal sludge at the Lavender Hill waste treatment facility in Accra [33]. Its closeness to Accra satisfied the Tricycle surveillance protocol’s requirement of being in proximity to the capital city (2–4 h required for transport of samples to the laboratory).

The Okurudu River lies between two river basins: Ayensu towards the west and Densu towards the east. It flows through the city and discharges into Nyanyano lagoon, an outlet into the Atlantic Ocean.

Upstream water samples from the Okurudu River were taken seven kilometres above the downstream sampling point. Communal waste (human wastewater) was sampled from an open drain located adjacent to a market characterised by heavy human activities, especially during its two market days. Animal waste was collected from a drain at a slaughter slab that deals in cattle, sheep and goats, while downstream samples were collected about seven kilometres away from the slaughter slab. Sampling points are shown on the map in Figure 1.

### 2.3. Sample Site Selection

Selection of sampling sites conformed to the WHO ESBL-Ec Tricycle Protocol [28] as shown in Table 1.

### 2.4. Sample Collection Period

Water samples were collected for a total of 12 months, broken into three periods. August–December 2018, October–February 2020 and May, June 2020 partly due to COVID-19 restrictions.

### 2.5. Sampling Method

Samples were collected between 6.00 a.m. to 12:00 p.m. At each sampling point, composite water samples were taken with a bailer; three scoops per sample, 20–30 cm below the water surface, five minutes apart. Samples were immediately transferred into sterile Nalgene 500 mL bottles after each scoop. Each sample was given a unique identifier for traceability. Ambient water temperature, air temperature, and pH (Multi-Parameter PCSTestr^TM^ 35 Eutech Instruments OakTon) of the samples were recorded along with the sample location, and date (month and year) of collection. Water colour, flow rate and turbidity were also rated and scored. Details of each sample were entered immediately onto a field sampling sheet after which samples were stored in a cold chest at 4 °C. All samples were transported to the laboratory and the information on each sample was entered into a Results Book at the laboratory. To access the laboratory in time for analysis to be carried out before 12 h, each city was sampled on alternate days. Sampling methods also conformed to methods outlined in Standard Methods for the Examination of water and wastewater [37] and ISO 19458:2006IDT, Water quality-sampling for microbiological analysis [38].

All field sampling and laboratory analysis procedures were carried out at the microbiology unit of the Council for Scientific and Industrial Research-Water Research Institute (CSIR-WRI).

### 2.6. Laboratory Procedures

In the laboratory, samples were mixed vigorously and diluted serially 10-fold using sterile Phosphate Buffered Saline solution. Suitable dilutions were vortexed and filtered through sterile 0.45 µm pore size membrane filters (MF-Millipore™ cellulose nitrate Membrane Filter). Filters were subsequently placed on both Tryptone Bile X-glucuronide medium (TBX) (Oxoid) and TBX supplemented with 4 μg/mL cefotaxime (TBX/CTX) and incubated at 37 °C for 18–24 h [37]. Plates with counts less than or equal to 100 blue-green colonies recovered from both (TBX) (Oxoid) and TBX/CTX were counted with the aid of a colony counter (Stuart^®^ SC6PLUS Digital Colony Counters, Stone, Staffordshire ST15 OSA (UK), United Kingdom). Five to eight cefotaxime resistant colonies from TBX supplemented media were streaked first for purity on TBX agar and subsequently on Nutrient Agar (oxoid) after 24 h. Suspected *E. coli* isolates were further confirmed by Indole-testing using Kovacs reagent. Cefotaxime-resistant *E. coli* were screened phenotypically for ESBL-production using the double-disc diffusion method according to Clinical and Laboratory Standards Institute (CLSI, 2016, Wanye, Pennsylvania, USA using Cefotaxime/Clavulanic Acid 30/10 μg, Cefotaxime 30 μg, Ceftazidime 30 μg, and Ceftazidime/Clavulanic Acid 30/10 μg (Becton Dickenson^TM^) [39]. Ratio ESBL-Ec over *E. coli* were calculated following ISO 8199 Water Quality-General guidance on the enumeration of micro-organisms by culture [40].

### 2.7. Quality Control Measures

All media were prepared according to the manufacturer’s instructions and the Tricycle Protocol. Each batch of media was pre-tested for sterility and the ability to support the growth of *E. coli*. Media and antibiotics were quality controlled using *E. coli* ATCC 25922, and *Klebsiella pneumoniae* ATCC 70063.

### 2.8. Data Collection, Source of Data and Validation

Study data were extracted from the Results Book of the Tricycle Project in the microbiology laboratory of CSIR-WRI and double entered into the study Excel database to ensure quality.

### 2.9. Statistical Analysis

Descriptive analysis was performed to show variations in the concentration of *E. coli*, ESBL-Ec and the percent ESBL-Ec/*E. coli* among all sampling sites. Normality of each dependent variable was tested using the Shapiro Wilk test. The Kruskal Wallis Sign Rank test was used to compare means of bacteria recovered among all four sampling sites while the Mann-Whitney U test was used to compare variations in bacterial concentration between the two cities. Physical parameters (pH and temperature) at the study locations of the two cities were compared using the independent sample t-test with cities used as grouping variables. Levene’s test was used to test for the equality of variance. All tests of statistical significance were set at a *p*-value of 0.05. To test for statistically significant association between temperature, pH and bacterial count, Spearman’s rank correlation was employed as bacterial data did not follow a normal distribution. The Statistical Package for Social Science (SPSS) software (IBM version 26) was used to perform all data analysis.

## 3. Results

Over the 12-month study period, 96 surface water samples were collected from the Odaw River located in Accra and the Okurudu River located in Kasoa.

### 3.1. Physical Characteristics of Water Samples

Overall, the mean ambient surface water temperatures in Accra ranged from 23.2 °C to 32.9 °C and in Kasoa from 25.8 °C to 35.2 °C. The pH levels of water samples in Accra ranged from 6.68 to 8.70 and in Kasoa 6.32 to 8.40.

When ambient water temperatures at each sample location were compared separately, variation was not significantly different among (1) downstream samples (*p* = 0.277) (Accra; Mean = 27.8 ± 2.1 °C, Kasoa; Mean = 28.8 ± 2.3 °C), (2) animal waste water samples (*p* = 0.098) (Accra; Mean = 29.3 ± 2.0 °C Kasoa; Mean = 29.5 ± 2.76 °C) and (3) human wastewater samples (*p* = 0.100) (Accra; Mean = 28.0 ± 2.5 °C, Kasoa; Mean = 30.0 ± 3.2 °C). Upstream water samples in both cities, however, showed significant variation in their ambient surface water temperatures (*p* = 0.005) (Accra; Mean = 26.85 ± 1.2 °C, Kasoa; Mean = 29.68 ± 2.7 °C).

When ambient water pH at each sample location was compared, there were no significant differences among (1) downstream samples (*p* = 0.107) (Accra; Mean = 7.76 ± 0.22, Kasoa; Mean = 7.92 ± 0.18), (2) animal waste water samples (*p* = 0.092) (Accra; Mean = 7.87 ± 0.48, Kasoa; Mean = 7.43 ± 0.63) and (3) human wastewater samples (*p* = 0.092) (Accra; Mean = 7.87 ± 0.49, Kasoa; Mean = 7.45 ± 0.63). Upstream water samples of both cities, however, showed significant differences in ambient water pH values (*p* = 0.012) (Accra; Mean = 7.60 ± 0.49, Kasoa; Mean = 8.07 ± 0.28).

### 3.2. E. coli Concentrations (cfu/100 mL) in Water Samples in Accra and Kasoa

**Figure 2:**
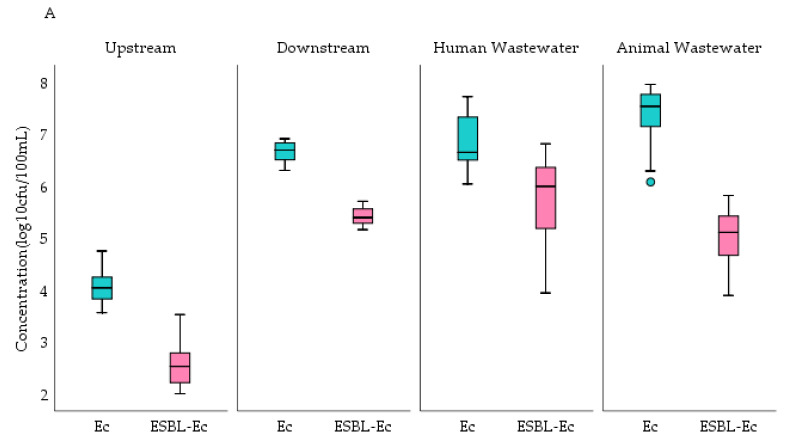

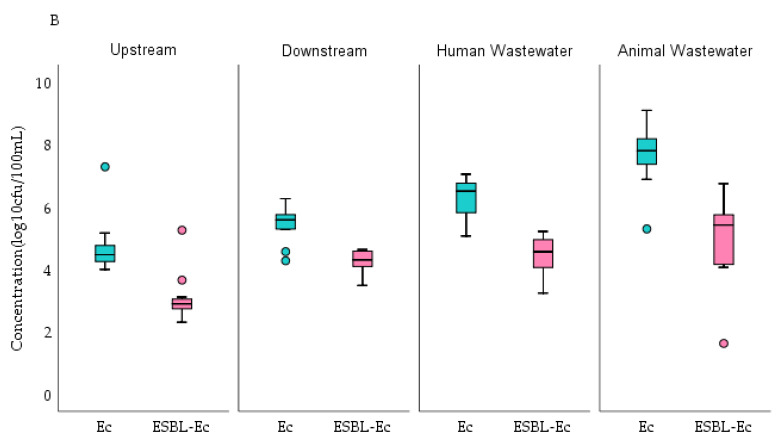
Comparison of concentrations of *E. coli* and ESBL-Ec among sampling sites in Accra (**A**) and Kasoa (**B**) for the periods of August 2018–December 2019, October 2019–February 2020, May, and Jun. 2020, Ec = *E. coli*, ESBL-Ec = Extended-Spectrum Beta-Lactamase *E. coli*, cfu = colony forming units.

**Figure 3:**
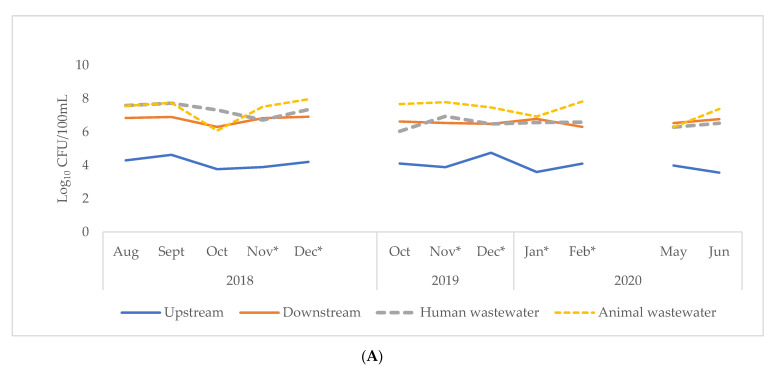

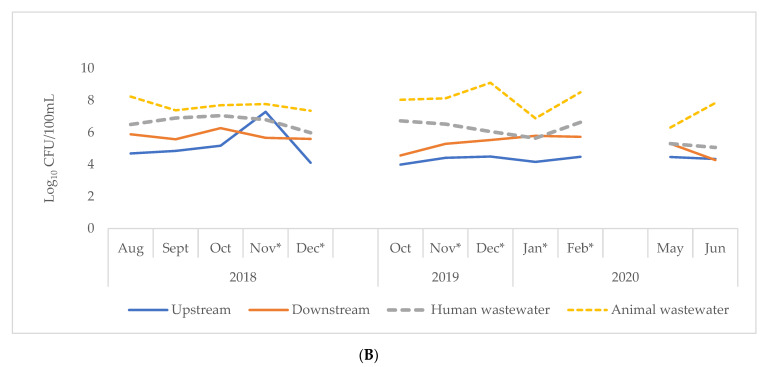
Log concentration of *E. coli* in sample sites of Accra (**A**) and Kasoa (**B**) for the periods: August 2018–December 2019, October 2019–February 2020, May, June 2020. * Indicates dry season, cfu = colony forming units.

Figure 2 shows *E. coli* concentration per 100 mL compared with ESBL-Ec per 100 mL of sample water collected in Accra and Kasoa. Overall, animal waste collected from both cities had the highest *E. coli* concentration of 4.8 (IQR, 2.4–8.5) × 10^7^ cfu/100 mL whilst upstream river samples had the lowest concentration of 1.8 (IQR, 1.0–3.9) × 10^4^ cfu/100 mL (Table 2). These observations were similar for both cities. Accra, however, had a higher *E. coli* concentration of 4.0 × 10^6^ (IQR, 3.2 × 10^5^–6.8 × 10^7^) when all samples were considered together, but this was not statistically significant when compared to Kasoa: 5.6 × 10^5^ (IQR, 8.0 × 10^4^–7.7 × 10^6^) (*p* = 0.23) (Table 2). Upstream *E. coli* concentrations were significantly greater in Kasoa at 2.9 (IQR, 1.9–5.3 × 10^4^) compared to Accra 1.1 × 10^4^ (IQR, 7.2 × 10^3^–1.7 × 10^4^) (*p* = 0.01). In contrast, significantly greater *E. coli* concentrations were found from Accra’s downstream 5.0 × 10^6^ (IQR, 3.3 × 10^6^–6.7 × 10^6^) and human 4.5 × 10^6^ (IQR, 3.2 × 10^6^–2.1 × 10^7^) (*p* = 0.04) wastewater samples compared to those of Kasoa’s downstream: 3.8 × 10^5^ (IQR 2.0 × 10^2^–5.4 × 10^5^), and human: 5.4 × 10^5^ (IQR, 8.2 × 10^5^–5.5 × 10^6^) (*p* = 0.00) wastewater (Figure 2). There were no significant differences in *E. coli* concentrations among animal wastewater samples in the two cities (*p* = 0.16). Throughout the study period, *E. coli* concentrations from each sampling point in both cities remained relatively constant as shown in Figure 3. There were no significant differences in bacterial counts between the wet and dry seasons.

There was a weakly positive correlation between water temperature and *E. coli* concentrations (ρ = 0.248) significant at *p* = 0.015 and a weakly negative correlation between pH and *E. coli* counts (ρ = −0.355) at a significant figure of *p* = 0.001.

### 3.3. ESBL-Ec Concentrations (cfu/100 mL) in Water Samples in Accra and Kasoa

**Figure 4:**
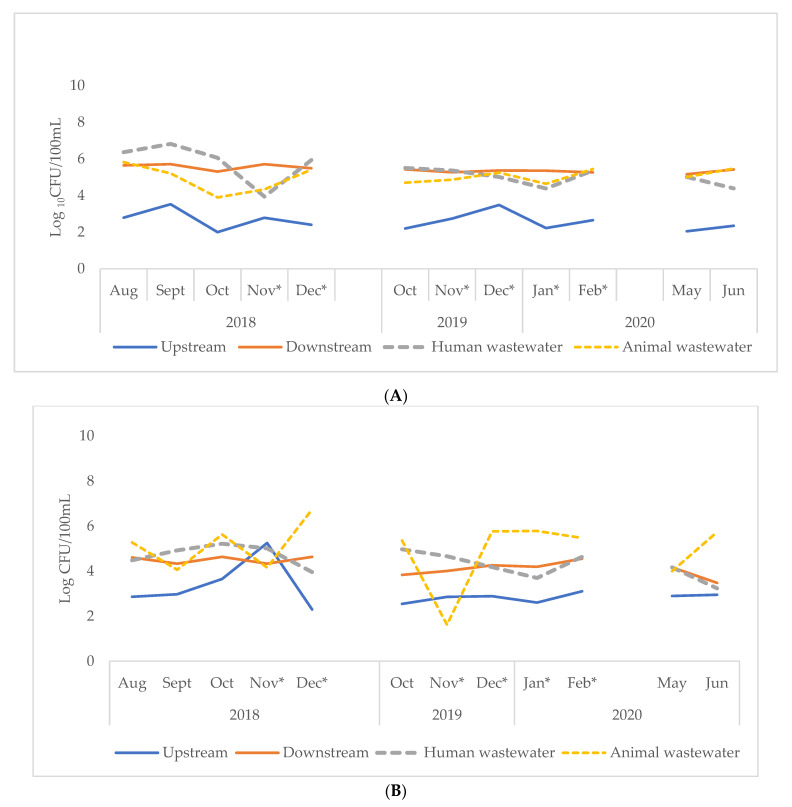
Log concentration of ESBL-Ec in sampling sites in Accra (**A**) and Kasoa (**B**) for the periods: August 2018–December 2019, October 2019–February 2020, May, June 2020. * Indicates dry season, ESBL-Ec = Extended-Spectrum Beta-Lactamase *E. coli*, CFU = colony-forming units.

ESBL-Ec were present in almost all samples analyzed-94 (98%). When sampling points from both cities were considered collectively, animal wastewater had the highest concentration of ESBL-Ec per sample at 1.8 (IQR, 0.3–3.9) × 10^5^ cfu/100 mL whilst upstream surface water samples had the least concentration at 6.0 × 10^2^ (IQR, 2.3 × 10^2^–9.2 × 10^2^) cfu/100 mL (Table 3). The ESBL-Ec concentration from each sampling point followed a similar pattern over the study period in both cities (Figure 4). However, significantly higher concentrations of ESBL-Ec were observed in samples from Accra: 1.7 × 10^5^ (IQR, 4.4 × 10^3^–3.0 × 10^5^) compared to Kasoa: 1.5 × 10^4^ cfu /100 mL (IQR, 2.0 × 10^3^–9.0 × 10^4^) (*p* = 0.03) (Table 3).

The ESBL-Ec concentrations were significantly higher (*p* = 0.03) in upstream waters of Kasoa: 7.8 (IQR, 6.4–10) × 10^2^ compared to upstream waters in Accra: 3.5 (IQR, 1.6–6.1) × 10^2^, (*p* = 0.03) (Figure 2). ESBL-Ec concentrations however, were significantly higher (*p* = 0.00) in downstream: 2.5 (IQR, 2.0–3.3) × 10^5^ and in human wastewater: 4.2 (IQR 0.8–9.3) × 10^5^ in Accra compared to Kasoa: downstream: 2.0 (IQR, 1.4–3.7) × 10^4^ and human wastewater: 3.7 (IQR, 1.3–8.6) × 10^4^ (Figure 2). There were no significant differences in ESBL-Ec concentrations between animal wastewater samples from both cities. (Figure 2). ESBL-Ec correlations with temperature and pH were not statistically significant.

In all, there was a strong positive association between *E. coli* and ESBL-Ec counts in cfu/100 mL (r = 0.761, *p* = 0.000).

### 3.4. Percentage ESBL-Ec (cfu/100 mL) Concentrations in Water Samples in Accra and Kasoa

**Figure 5:**
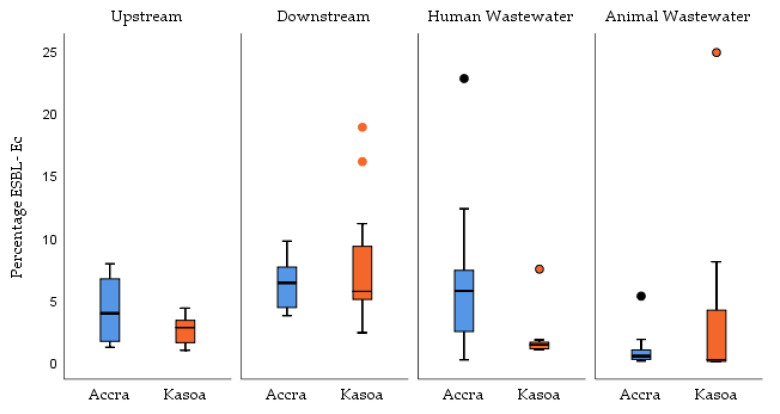
Comparison of percent ESBL-Ec in Accra and Kasoa or the periods of August 2018–December 2019, October 2019–February 2020, May, and June 2020, ESBL-Ec = Extended-Spectrum Beta-Lactamase *E. coli*.

**Figure 6:**
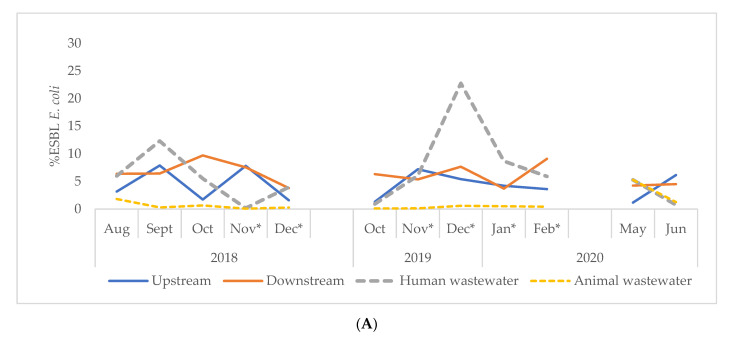

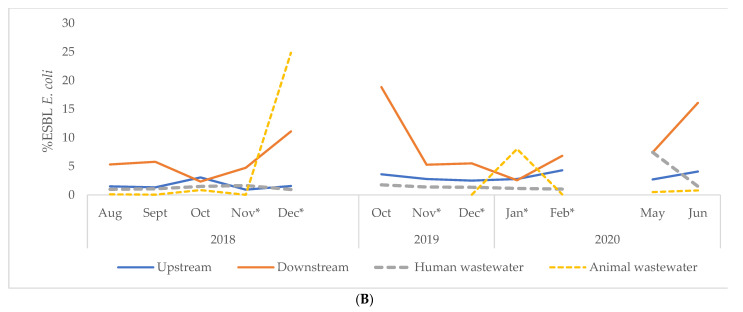
Proportion of ESBL-Ec among all *E. coli* in sites in Accra (**A**) and Kasoa (**B**) for the periods of August 2018–December 2019, October 2019–February 2020, May and June 2020. * Indicates dry season, ESBL-Ec = Extended-Spectrum Beta-Lactamase *E. coli*.

Figure 5 shows the proportion of ESBL-Ec among the total number of *E. coli* isolated per sample from Accra and Kasoa. Overall, the proportion of ESBL-Ec/*E. coli* isolates per sample from both cities was 2.79% (IQR, 1.04–6.03) (Table 4). The highest proportion of ESBL-Ec was obtained from downstream water samples at 6.06% (IQR, 4.57–7.64), and the lowest was from animal wastewater at 0.30% (IQR, 0.10–0.84) (Table 4). Upstream samples had a relatively lower percentage ESBL-Ec/Ec at 2.93% (IQR, 1.58–4.29) compared to downstream water samples. Samples from Accra generally had a higher proportion of ESBL-Ec at 4.08% (1QR, 0.78–6.37) compared to Kasoa at 2.06% (IQR, 0.99–5.32). Over the study period, the proportion of ESBL-Ec isolated per sample fluctuated significantly across both study sites (Figure 6). There was a weak negative correlation between temperature and percentage ESBL-Ec (−0.294) at a significant value of *p* = 0.004 and a weak positive correlation between pH and percentage ESBL-Ec (0.215) at a significant value of (0.044).

## 4. Discussion

In rivers flowing through two cities in Ghana, we showed high concentrations of *E. coli* and relatively low but significant concentrations of ESBL-Ec cfu/100 mL from all water samples collected. The counts increased significantly from upstream to downstream, indicating that considerable contamination was taking place as the rivers flowed through the two cities.

In both Accra and Kasoa, almost all water samples recorded *E. coli* levels significantly above WHO guideline thresholds: ≤10^4^ *E. coli* counts per 100 mL are allowed for restricted irrigation of crops that require labour-intensive high contact agriculture and ≤10^3^ for un-restricted irrigation of root crops that may be eaten uncooked [41]. Counts were also much higher than Ghana’s standard limits (*E. coli* ≤10 cfu/100 mL) for discharge of wastewater into the environment [42]. *E. coli* concentrations in downstream water (4.3 (IQR, 3.5–4.7) log_10_ cfu/mL) were slightly higher than those found from downstream water in South Africa (3.6–3.8 log_10_ cfu/mL) [43], and lower compared to surface water in Kanpur, India (5.1 log_10_ cfu/mL) [26].

In our study, almost all samples, i.e., 98%, were positive for ESBL-Ec. Other studies have shown relatively lower ESBL-Ec recovery rates of 79.7% from untreated wastewaters in Nigeria [21], 28.6% from wastewater and recipient surface waters from a treatment plant in South Africa [43], 64% from environmental water sources in India and 59% from different aquatic sources in Nicaragua [44].

In our study, increased contamination of downstream by and ESBL-Ec compared to upstream water was probably due to wastewater discharges into the rivers in populated areas [43]. In a recent study in Accra that assessed faecal exposure by comparing variation in *E. coli* counts from urban open drains of four neighborhoods, the *E. coli* concentrations were lower in improved (>85%) sanitation coverage neighborhoods with high-income and lower population density and higher in poor (<50%) sanitation coverage neighborhoods with low-income and higher population density. *E. coli* counts (>4 log10 CFU/100 mL) recovered from the two population categories are enough to pose a public health hazard [45]. In Ghana, like most LMICs, drainage systems consist of street gutters and storm drains that are not covered, making them very prone to runoff from all sources [46]. The absence of functioning sewer drainage systems in most homes results in untreated greywater and black water from septic tanks being discharged into the environment [47].

While there were no differences in *E. coli* concentrations between the two cities, the concentration of ESBL-Ec per 100 mL of sample in Accra was 2.5 log units higher than in Kasoa. These differences could be due to two causes. First, there may be higher levels of community carriage of ESBL-Ec among the population of the catchment area in Accra, presumably from human and animal waste that is discharged without treatment into the rivers. For example, high carriage of ESBL-Ec among humans was found in Korle-Gonno-96.4% (134/139), a suburb of Accra with drains connected to the Odaw River [16]. Second, the rivers themselves could have differing concentrations of ESBL-Ec before entering the cities. In fact, the proportion of ESBL-Ec to *E. coli* was already higher in the upstream location of Accra as compared to Kasoa, as was the proportion of ESBL-Ec in human waste. It is also expected that Accra, being a larger city with more human and animal wastewater, would have a higher count of ESBL-Ec in its downstream waters than Kasoa.

Information on risk factors for ESBL-Ec carriage is mostly available for highly developed countries and includes consumption of antibiotics and recent hospitalisation [48]. It is likely that antibiotic use is also a determinant for ESBL-Ec carriage in Ghana; however, how it compares to other risk factors like human–animal contact or travel to other countries remains to be determined. ESBL-Ec can easily be transmitted between household members, so higher ESBL-Ec proportions might be maintained by higher population densities or tighter housing conditions [49]. Finally, an increased risk for ESBL-Ec acquisition during treatment of children with malnutrition has been found during amoxicillin therapy in Niger so the degree of malnourishment in the population might affect overall ESBL-Ec carriage [50]. The difference in ESBL proportions between the more urbanized Accra region and Kasoa also deserves further attention.

There were large variations in proportions of ESBL-Ec among *E. coli* from both cities 2.79 (IQR, 0.96–6.03%). This was comparable to variations in the proportion of ESBL-Ec (0.1–19%) from a French community effluent [51]. Among sampling points, the highest percentage of ESBL-Ec was found in the downstream water while the lowest was found in the animal wastewater. Thus, other sources, including human waste, probably contribute to increased ESBL-Ec presence in the downstream samples. The low proportions of ESBL-Ec concentrations among *E. coli* seen in this study are similar to other contexts such as 1.7% that has been found in a city wastewater, 0.2% in a slaughterhouse wastewater, 0.2% in a treated effluent in France [52] and 1% from a constructed wetland also in France [53]. Occurrence of AMR bacteria in sub-quantities is known to create ideal conditions for bacteria to fix genes and to allow for gene amplification [54] and should not be underrated.

Generally, climatic conditions (temperature and pH) in both cities during the study period were conducive for bacteria growth. The prevailing pH of ambient water provides an optimum growth range for most bacteria and falls within the USEPA Recommended Water Quality Criteria for Aquatic Life (6.5–8.5) [55]. *E. coli* has been shown to have a significant negative correlation with pH in water columns [56]. Though consistent with our findings, our correlation of *E. coli* with pH was not significant. Greater counts of bacteria recovered in the upstream water of Kasoa compared to Accra suggests relatively better protection of the source waters of Accra compared to Kasoa. Removal of riparian vegetation causes an increase in temperature of water and sediments and this facilitates coliform and *E. coli* survival and growth [57]. In general, the occurrence of coliform bacteria are significantly higher when water temperatures are above 15 °C [58]. However, temperature overall only had a modest effect on absolute concentrations of ESBL-Ec. in our study. There were also no significant differences in bacterial counts between the wet and dry seasons, as has been shown by other studies [59,60].

Ghana is one of the pilot countries in which the Tricycle Protocol was investigated with respect to its feasibility. Our results showed that the Protocol could easily be established in the CSIR-WRI laboratory and that it was clear, sufficiently detailed and specific. The laboratory’s experience of *E. coli* testing as a water quality indicator and methods for its quantification (specifically, membrane filtration) helped the implementation of the Tricycle methodology. The Protocol is targeted towards implementation under circumstances without the presence of advanced laboratory infrastructure (e.g., MALDI-TOF for confirmation of species obtained) but nevertheless is expected to give results of high specificity through the use of assays based on the beta-glucuronidase activity of *E. coli*, which have a high sensitivity and specificity for determining *E. coli* concentrations in water. [61,62].

The strengths of this study were: first, we followed standardised methods for collecting and analysing the water samples. Second, all water samples collected were accounted for in the results. Third, the laboratory where the samples were processed is routinely monitored by the Eurofins proficiency testing scheme [63]. Finally, the conduct and reporting of the study adhered to Strengthening The Reporting of Observational Studies in Epidemiology (STROBE) guidelines [64].

There were some limitations of the study. First, more sampling sites could have been chosen to increase the precision of the location of ESBL-Ec contamination. Second, the breaks in the planned 12-month continuous testing plan resulted in the testing being carried out in three sections. This meant that variations due to wet and dry seasons could not be demonstrated. Third, measuring additional physicochemical parameters during sampling events may have given more clues to the growth of resistant *E. coli* as temperature and pH are not the only such influences on bacterial growth. Fourth, a full profile of antimicrobial susceptibility testing was not done that would have highlighted the level of multi-drug resistance among these organisms. Fifth we did not take into consideration the possible occurrence of glucuronidase negative *E. coli* as we dealt mainly with wastewater and not potable water samples.

This study has several implications. It has shown presence of ESBL-Ec in water bodies with the potential to transmit to humans and animals. Hitherto, the strategy for controlling AMR has focused on managing the use of antibiotics in humans and animals. This study points to environmental contamination as a potential source for AMR and suggests transmission related to such environmental contamination should be explored further. A relevant follow-up study would be to perform genetic analysis of the ESBL-Ec types found in humans and animals to see whether they correlate with types observed in the river.

Reducing environmental contamination could be achieved through improvements of the basic sanitation infrastructure and in household toilets for the population to reduce the practice of open defecation. It should also include improving the number and efficiency of sewage treatment plants. However, given limited budgets, this is unlikely to happen soon. In the short term, there is an urgent need to educate the population living around the rivers regarding the risks of using that water for domestic purposes and the need to render it safe before its use. Education should focus on reducing the practice of discharging untreated waste into water bodies.

## 5. Conclusions

This study revealed high levels of *E. coli* and a significant concentration of ESBL-Ec in rivers flowing through two cities in Ghana. Both counts increased during their passage through the cities indicating contamination by human and animal wastewater. This situation may contribute to the spread of AMR among humans and animals in Ghana and should be considered a factor for further study. The Tricycle Protocol demonstrated its feasibility in a context like Ghana and the data gathered will serve as a good baseline for comparison with future studies.

## Figures and Tables

**Table 1:** ESBL-Ec Tricycle Protocol.

	Summaries of Study Elements
Number of Cities	Within One Country: Two Cities Should be Identified
Eligible City	Major (capital) city where the analysis laboratories are located. This city must have a hospital health care facility and a wet market for the study of the two other Tricycle work packages; WP1 (ESBL-Ec in food chain) and WP2 (ESBL-Ec in humans). As well as a sentinel city of about 100,000 inhabitants, in proximity to the capital city. Both require the presence of a river.
Who helps with the selection of sites	Within each city, the identification of collection sites for each sample type will be performed with the assistance of other authorities and specialists to identify the most representative sampling sites.
Sample points	Four sample points from each city: Two hotspot sources, an upstream and a downstream point on rivers receiving wastewater from these cities. River water upstream of the city, representing pre-city impacts and other upstream activities in the catchment area—serves as a reference sample to detect the influence from the city. River water downstream of the city is representative of city impacts Communal waste (influent of a treatment plant, or major collecting sewers) representing human faecal material. Waste from a wet market (or slaughterhouse, if wet markets are not present), representing animal faecal material.
Sampling rounds	Monthly sampling for one year. Number of samples: 2 cities × 4 samples × 8–12 rounds/year = 64–96 samples.
Analysis parameters	Concentration of *E. coli* Concentration of ESBL producing *E. coli* (ESBL-Ec) Ratio ESBL-Ec over *E. coli*

**Table 2:** Concentrations of *Escherichia coli* (Ec), stratified by site, city, and season in water samples from Accra and Kasoa, Ghana, from August 2018–December 2019, October 2019–February 2020, May, and June 2020.

	Sample Type	Sample Number	Median Concentration	(Q1 ^b^, Q2 ^c^)	
		(N)	(Ec ^a^ cfu/100 mL × 10^7^)	cfu/100 mL × 10^7^	P ^d^
Sites					0.00
	Upstream	24	0.0018	(0.00096, 0.0039)	
	Downstream	24	0.19	(0.037, 0.54)	
	Human wastewater	24	0.37	(0.13, 0.84)	
	Animal wastewater	24	4.8	(2.4, 8.5)	
					0.86
Season	Wet	48	0.21	(0.0053, 0.21)	
	Dry	48	0.32	(0.019, 0.86)	
City					0.23
	Accra	48	0.40	(0.032, 2.3)	
	Kasoa	48	0.06	(0.008, 0.77)	
	Sample Total	96	0.3	(0.008, 1.8)	

^a^ Ec = *Escherichia coli*, ^b^ Q1 first quartile, ^c^ Q3 third quartile, ^d^ Mann-Whitney U/Kruskal Wallis test, CFU = colony forming units.

**Table 3:** Concentrations of ESBL-Ec ^a^, stratified by site, city and season of water samples from Accra and Kasoa, Ghana, from August 2018–December 2019, October 2019–February 2020, May, and June 2020.

	Sample Type	Sample Number	Median Concentration	(Q1 ^b^, Q2 ^c^)	
		(N)	(ESBL-Ec) ^a^ cfu/100 mL × 10^4^	cfu/100 mL × 10^4^	P ^d^
Sites					0.00
	Upstream	24	0.061	(0.023, 0.092)	
	Downstream	24	9.30	(1.90, 25)	
	Human wastewater	24	8.80	(1.50, 47)	
	Animal wastewater	24	18	(2.7, 39)	
Season					0.94
	Wet	48	3.5	(0.31, 25)	
	Dry	48	4.3	(0.35, 23)	
City					0.03
	Accra	48	17	(0.44, 30)	
	Kasoa	48	1.5	(0.20, 9.0)	
	Sample Total	96	4.2	(0.31, 23)	

^a^ ESBL-Ec—Extended Spectrum Beta-lactamase *Escherichia coli*, ^b^ Q1 first quartile, ^c^ Q3 third quartile, ^d^ Mann-Whitney U/Kruskal Wallis test, CFU = colony-forming units.

**Table 4:** %ESBL-Ec, stratified by site, city and season of water samples from Accra and Kasoa, Ghana, from August 2018–December 2019, October 2019–February 2020, May, and June 2020.

	Sample Type	Sample Number	Median Proportion	(Q1 ^b^, Q2 ^c^)	
		(N)	(%ESBL-Ec) ^a^	(cfu/100 mL)	P ^d^
Sites					0.00
	Upstream	24	2.93	(1.57, 4.29)	
	Downstream	24	6.06	(4.57, 7.64)	
	Human wastewater	24	1.58	(1.04, 6.00)	
	Animal wastewater	24	0.30	(0.10, 0.84)	
Season					1.00
	Wet	48	2.53	(1.00, 6.10)	
	Dry	48	3.20	(0.93, 6.01)	
**City**					0.07
	Accra	48	4.08	(0.79, 6.37)	
	Kasoa	48	2.06	(0.99, 5.32)	
	Sample Total	96	2.79	(0.96, 6.03)	

^a^ ESBL-Ec—Extended Spectrum Beta-lactamase *Escherichia coli*, ^b^ Q1 first quartile, ^c^ Q3 third quartile, ^d^ Mann-Whitney U/Kruskal Wallis test, CFU = colony-forming units.

## Data Availability

Data is available on request.
